# Targeted Therapy for Hepatocellular Carcinoma: Old and New Opportunities

**DOI:** 10.3390/cancers14164028

**Published:** 2022-08-20

**Authors:** Carmelo Laface, Palma Fedele, Felicia Maria Maselli, Francesca Ambrogio, Caterina Foti, Pasquale Molinari, Michele Ammendola, Marco Lioce, Girolamo Ranieri

**Affiliations:** 1 Medical Oncology, Dario Camberlingo Hospital, 72021 Francavilla Fontana, BR, Italy; 2 Section of Dermatology, Department of Biomedical Science and Human Oncology, University of Bari, 70124 Bari, Italy; 3 IRCCS Istituto Tumori “Giovanni Paolo II”, 70124 Bari, Italy; 4 Department of Health Science, General Surgery, Medicine School of Germaneto, Magna Graecia University, 88100 Catanzaro, Italy

**Keywords:** hepatocellular carcinoma, cancer therapy, targeted therapy, tyrosine kinase inhibitors, immunotherapy, immune checkpoint inhibitors

## Abstract

**Simple Summary:**

Hepatocellular carcinoma (HCC) is the most frequent primitive cancer of the liver, accounting for 90% of all recorded cases. HCC is the third most common cause of cancer-related death, with a 5-year survival rate of just 3%. In terms of the advanced stages, systemic treatments have allowed patients to achieve clinical benefits, although the prognosis remains very poor. In the past few decades, new molecular targeted therapies have been developed and clinically evaluated with interesting results. However, on the basis of the poor prognoses and the meager benefits deriving from the available systemic therapies, research into new treatments is extremely necessary. In this review, we focus on the available systemic therapies for advanced HCC, with a look toward the future.

**Abstract:**

Hepatocellular carcinoma (HCC) is the most frequent primitive cancer of the liver, accounting for 90% of all recorded cases. HCC is the third most common cause of cancer-related death, with a 5-year survival rate of just 3%. In the advanced stages, systemic treatments allow doctors to obtain clinical benefits, although the prognosis remains very poor. In the past few decades, new molecular targeted therapies against receptor tyrosine kinases have been developed and clinically evaluated. Sorafenib was the first oral tyrosine kinase inhibitor (TKI) approved for the treatment of advanced HCC in 2007. Subsequently, other TKIs, including Cabozantinib, Regorafenib, Lenvatinib, and vascular endothelial growth factor receptor (VEGFR) inhibitors such as Ramucirumab and VEGF inhibitors such as Bevacizumab have been approved as first- or second-line treatments. More recently, the combination of immune checkpoint inhibitors and VEGF inhibitors (Atezolizumab plus Bevacizumab) have been analyzed and approved for the treatment of advanced HCC. On the basis of the poor prognoses and the meager benefits deriving from the available systemic therapies, research into new treatments is extremely necessary. In this review, we focus on the available systemic therapies for advanced HCC, with a look toward the future.

## 1. Introduction

Hepatocellular carcinoma (HCC) is the most frequent primitive cancer of the liver, accounting for 90% of all cases [1]. It is considered the sixth most common cancer worldwide and its incidence has been progressively increasing. HCC is also the third most common cause of cancer-related death, with a 5-year survival rate of just 3% [2]. Therefore, it is seen as a major public health problem around the world.

HCC treatment mainly depends on the tumor stage. Nowadays, numerous staging systems have been designed; however, the Barcelona Clinic HCC (BCLC) system is the most famous and is commonly employed in clinical practice [3]. It is based on liver function (Child–Pugh score), tumor size and number, and performance status (PS) [3,4]. The BCLC staging system allows doctors to manage HCC patients, in terms of prognosis and treatment [3,4].

Liver resection, liver transplantation, and loco-regional therapies such as ablation with radiofrequency (RFA), transarterial radioembolization (TARE), or chemoembolization (TACE) correspond to the gold standard treatments for early stages [5,6,7,8,9,10]. However, 70% of these patients experience a recurrence of the disease in the following 5 years. In addition, only 30% of HCCs are diagnosed in the early stages, due to the silent clinical history of this disease [2].

As regards the advanced stages, systemic treatments are the only therapies available that obtain a clinical benefit, although the prognosis remains very poor. Therefore, the development of new systemic therapeutic options is necessary to improve the clinical outcomes of advanced HCC patients. In the last few decades, new molecular-targeted therapies have been developed. To be specific, several small molecules targeting receptor tyrosine kinases (RTKs) have been clinically evaluated. Sorafenib was the first oral multi-tyrosine kinase inhibitor (TKI) approved for the treatment of advanced HCC in 2007, demonstrating an improvement in the survival of these patients [11]. Subsequently, other TKIs, including Cabozantinib, Regorafenib, Lenvatinib, and vascular endothelial growth factor receptor (VEGFR) inhibitors such as Ramucirumab and VEGF inhibitors such as Bevacizumab have been approved in this setting of patients, as first- or second-line treatments [12]. In the last years, immune checkpoint inhibitors (ICIs), the anti-programmed cell death-1 (anti-PD-1) antibodies, have been tested and have obtained approval from the Food and Drug Administration (FDA) [13,14,15]. More recently, the combination of ICIs and VEGF inhibitors (Atezolizumab plus Bevacizumab) has been analyzed and approved for the treatment of advanced HCC [16,17].

Based on the poor prognoses and the meager benefits derived from the available systemic therapies, research into new therapies is extremely necessary to address the challenge of HCC for clinicians. Interestingly, several preclinical and clinical studies are ongoing worldwide. In this review, we focus on the available systemic therapies for advanced HCC and look at the most recent developments in the field. In particular, we summarize the clinical results regarding the current therapies and discuss the challenges and new directions in the development of new systemic treatments.

## 2. Targeted Therapies

### 2.1. Tyrosine Kinase Inhibitors

Tyrosine kinases are a group of small proteins able to modulate cell differentiation and signaling [18]. To be specific, they act through phosphorylation, using the ATP phosphate groups of tyrosine residues of different proteins, including RTKs [19]. The phosphorylated RTKs become active and can transfer various signals through several intracellular signaling molecules within cells, regulating their growth, differentiation, and death. The most important signaling pathways involved are Ras/Raf/MEK/ERK and PI3K/AKT/mTOR [20,21,22].

VEGFR, epidermal growth factor receptor (EGFR), fibroblast growth factor receptor (FGFR), platelet-derived growth factor receptor (PDGFR), and insulin receptor (INsR) are examples of RTKs that are typically involved in tumorigenesis [18,23]. In fact, the mutated or abnormal expression of tyrosine kinases is often found in different types of cancers such as HCC, playing a pivotal role in tumor growth [23,24]. Therefore, preclinical and clinical research have documented the efficacy of TKIs in the treatment of several cancers [25,26,27,28,29,30,31,32,33,34,35,36]. With regard to HCC, the first TKI with proven efficacy was Sorafenib in 2007 [37]. Subsequently, other TKIs have been approved for the treatment of advanced HCC (Table 1).

#### 2.1.1. Sorafenib

Sorafenib (Nexavar) was the first oral multi-TKI to be approved for the treatment of advanced HCC in 2007 [37]. Before this date, no therapies were available for this type of tumor, so this drug has dramatically changed the natural history of advanced HCC.

Sorafenib has a dual pharmacodynamic action that is anti-proliferative and anti-angiogenic, through the blockage of Raf/MEK/ERK and JAK/STAT and the inhibition of phosphorylation of almost 40 tyrosine kinases, such as VEGFRs, PDGFR-β, c-Kit, FLT3, and RET [42]. In vitro studies demonstrated the anti-proliferative, anti-angiogenic, and pro-apoptotic activities of Sorafenib in HCC cell lines [43]. Preclinical studies in mouse models documented both anti-tumor and anti-metastatic effects [44,45]. Furthermore, the efficacy of Sorafenib seems due to its ability to target both tumor cells and the tumor microenvironment [46]. To be specific, experimental data evidenced Sorafenib’s impact on HSCs proliferation through the suppression of α-SMA- and PDGF-related pathways [47]. Moreover, it has been described that a high dose of Sorafenib could promote immunosuppression, inducing PD-1 expression in infiltrating immune cells [48].

The Sorafenib HCC assessment randomized protocol (SHARP) trial was a phase-3, multicenter, double-blinded placebo-controlled assay that evaluated the safety and efficacy of this drug for advanced HCC and its ability to provide an improvement in oncological outcomes [37]. Subsequently, Sorafenib became the first-line treatment for this set of patients. To be specific, the SHARP clinical trial enrolled 602 Western advanced HCC patients who had never been treated before with systemic treatments; almost half of them had never received any form of treatment for HCC. Child–Pugh Stage A disease was present in 97% of patients. Hepatitis C (29%), alcohol intake (26%), and hepatitis B (19%) were the most common etiologies of HCC. Finally, 51% of patients had an extrahepatic disease and 70% of them had a macroscopic vascular invasion.

The patients were randomly divided (in a 1:1 ratio) into the experimental group receiving Sorafenib (400 mg twice a day) and the control group receiving a placebo. Primary endpoints corresponded to OS and the time to symptomatic progression (TTSP), while secondary outcomes included the time to radiologic progression (TTRP) and safety. Statistical analysis evidenced a significant benefit in OS (10.7 versus (vs.) 7.9 months; hazards ratio (HR) = 0.69; *p* < 0.001) and TTRP (5.5 vs. 2.8 months; HR = 0.58; *p* < 0.001). Seven patients in the experimental group (2%) and two patients in the control group (1%) had a partial response; no complete response was observed. Disease control rate (DCR) was 43% vs. 32% (*p* = 0.002), respectively. The Sorafenib group experienced more grade-III drug-related adverse events (AEs) than the control group (8% vs. 2%; *p* < 0.001). The Forrest plot did not divide according to age, gender, or race.

The most common AEs were diarrhea, hand-foot skin reaction (HFSR), and hypertension. In a randomized study, Ren et al. demonstrated that urea-based creams could improve the HFSR-associated quality of life (QoL) during Sorafenib treatment [49]. In addition, a significant correlation exists between AEs and the OS of advanced HCC patients treated with Sorafenib [50,51]. Thus, HFSR may act as a clinical biomarker of Sorafenib efficacy.

As mentioned earlier, most of the patients enrolled in the SHARP trial were from Western countries; hepatitis C, alcohol intake, and hepatitis B were the most common etiologies of HCC. The Asian Pacific (AP) trial was a randomized double-blind placebo-controlled and evaluated the safety and efficacy of Sorafenib on 226 Oriental patients [38]. In addition, in the Asian Pacific region, the most frequent etiology of HCC is hepatitis B (71%). Child–Pugh Stage A disease was present in 97% of patients. Finally, 68% of patients had an extrahepatic disease and 35% of them had a macroscopic vascular invasion.

Sorafenib also improved survival benefit (6.5 vs. 4.2 months; HR=0.68; *p* = 0.014) and time-to-progression (TTP; 2.8 vs. 1.4 months; HR = 0.57; *p* = 0.0005) in this patient population. The DCR was significantly greater in the experimental group than in the control group (35% vs. 16%; *p* = 0·0019). Patients aged < 65 years benefited more from treatment. Severe AEs were reported in 9% of patients in the sorafenib group and 1% in the placebo group.

The OS appears greater in the SHARP trial than in the AP trial (10.7 vs. 6.5 months). For a better explanation of this data, different subgroups of patients were studied according to the various etiologies. Specifically, HBV patients were 19% in the SHARP trial and 71% in the AP trial; on the other hand, the HCV patients were 29% and 11%, respectively. Subgroup analyses showed that treatment with Sorafenib led to a better survival benefit in HCV patients compared to HBV ones [52,53]. Moreover, TTP was also significantly longer for HCV patients than for HBV ones (6.5 vs. 4.0 months, respectively, *p* = 0.05) [54]. In support of these data, a meta-analysis of Phase III trial results confirmed a survival benefit for patients with HCV etiology, compared to HBV [55]. Therefore, hepatitis may be a dependent risk factor for Sorafenib efficacy. Furthermore, a low neutrophil-to-lymphocyte ratio (NLR) and no extrahepatic spread of disease are predictive factors of a superior Sorafenib response [56].

Finally, the study showed that the greatest efficacy of Sorafenib was seen in those patients with a good liver function reserve, in Child–Pugh Stage A [57]. The treatment of patients with poor liver function, Child–Pugh Stage B, led to a smaller benefit in terms of survival and a higher incidence of AEs compared to Child–Pugh Stage A [58,59]. Therefore, although there is no limitation to the treatment of patients with Child–Pugh Stage B, these patients should be carefully followed during treatment, due to the higher risk of severe AEs [58,59].

#### 2.1.2. Lenvatinib

Lenvatinib (Lenvima) is another oral multi-TKI. It acts by inhibiting VEGFR1–3, PDGFR-α, FGFR1-4, KIT, and RET [60,61]. Lenvatinib showed anti-angiogenic and anti-FGFRs activities in mouse and in vitro models [62,63]. Furthermore, it has been reported that Lenvatinib exerts an immunomodulatory action by increasing the CD8^+^ T cell population and reducing monocytes and macrophages in HCC cells [64].

Lenvatinib has been analyzed as a first-line treatment in the REFLECT trial [39,65]. The latter is an open-label randomized phase-III non-inferiority clinical trial that compared Lenvatinib to Sorafenib as a frontline therapy for advanced HCC. The patient population consisted of 954 advanced HCC patients who had never been treated before with systemic treatment; 30% of them did not receive any prior regional treatment for HCC. Approximately one-third of patients were Westerners and almost all had good liver function (Child–Pugh Stage A). Hepatitis B and C (53% and 19%, respectively) were the most common etiologies. Two-thirds of patients had extrahepatic spread. Interestingly, the REFLECT trial excluded those patients with 50% or higher liver tumor burden, gross invasion of the bile duct, or invasion at the main portal vein.

No significant difference was observed between the Lenvatinib treatment group (*n* = 478) and the Sorafenib one (*n* = 476) in terms of OS (13.6 vs. 12.3 months; HR = 0.92), the primary endpoint, meeting the non-inferiority criteria. Otherwise, the experimental group experienced a higher objective response rate (ORR) (24.1% vs. 9.2%; *p* < 0·0001) and longer progression-free survival (PFS) (7.4 vs. 3.7 months; HR 0.66; *p* < 0.0001) and TTP (8.9 vs. 3.7 months; HR 0.63; *p* < 0·0001). Patients aged ≥ 65, who were female or Asian benefited more from this treatment.

Grade ≥ 3 AEs occurred at similar rates in the two groups (75% vs. 67%). More serious AEs were observed in the Lenvatinib group, including hypertension, proteinuria, anemia, dyspnea, thrombocytopenia, and hypothyroidism. Consequently, this could explain the higher percentage of treatment discontinuation in the experimental group (40% vs. 32%). Hypertension (42%) and diarrhea (39%) were the most frequent any-grade AEs in the Lenvatinib group, while palmar-plantar erythrodysesthesia (52%), diarrhea (46%), and hypertension (30%) were the most frequent AEs in the Sorafenib group.

Therefore, according to the reported results, Lenvatinib was approved as a first-line systemic treatment in advanced HCC.

Further statistical analyses showed that patients with hepatitis B and a high alpha-fetoprotein (AFP) level in serum (>200 ng/mL) showed a higher efficacy from Lenvatinib compared to Sorafenib [39,60,65]. Moreover, it has been reported that the levels of AFP decreased in the two weeks after the beginning of treatment [66]. This suggests that AFP levels might be a predictive factor of response to Lenvatinib. Moreover, circulating FGF-19 and Ang-2, as well as early tumor shrinkage, have also been proposed as predictive factors of clinical response to Lenvatinib in HCC patients [67,68].

In summary, Lenvatinib is not inferior to Sorafenib in OS, but it is superior in terms of secondary endpoints, such as ORR, PFS, and TTP [69]. The enrolled patient populations are different between the SHARP and REFLECT trials, in particular regarding liver tumor burden, gross invasion of the bile duct, or invasion at the main portal vein. As regards safety, Lenvatinib causes less diarrhea, fewer hand-foot skin reactions, and weight loss, while Sorafenib is better tolerated in terms of hypertension, hypothyroidism, and proteinuria [39]. Sorafenib has been present in clinical practice since 2007 so its use is more familiar, as well as the management of AEs. Finally, no clinical trials have been conducted on patients who progressed to Lenvatinib as a frontline therapy, unlike Sorafenib. Therefore, the choice between these drugs is at the clinicians’ discretion.

#### 2.1.3. Cabozantinib

Cabozantinib (Cometriq, Cabometyx) is another oral multi-TKI that targets VEGFR 1–3, MET, RET, KIT, TIE2, FLT3, c-MET, and AXL [70]. The blockage of c-MET and AXL is the most important pharmacodynamic difference compared to Sorafenib and Regorafenib because this allows Cabozantinib to overcome the resistance to these drugs [71]. In fact, it has been reported that MET and AXL receptors are involved in antiangiogenic resistance, as well as in epithelial-mesenchymal transition, invasion, and metastasis [70]. Therefore, Cabozantinib is also able to overcome Sorafenib and Regorafenib resistance, as confirmed also in clinical studies. Experimental data demonstrated Cabozantinib activity in the inhibition of tumor growth, angiogenesis, invasion, and migration [71]. Moreover, it also reduced the number of HCC lung and liver metastases in mouse models [46,71].

CELESTIAL is a randomized, double-blinded phase III clinical trial that tested Cabozantinib at 60 mg daily vs. placebo as a second-line therapy in 707 patients affected by advanced HCC patients who progressed to Sorafenib, who received at least one systemic treatment, or up to two previous systemic treatments [40]. To be specific, almost 70% of patients had undergone only Sorafenib before, while one-third of them had previously received two systemic treatments. Approximately 70% of enrolled patients were from Western countries; hepatitis B was present in 38% of patients while 25% suffered from hepatitis C. Almost 80% presented extrahepatic disease and one-third had macrovascular invasion [40].

The study reported a significant improvement for the experimental group in terms of OS (10.2 vs. 8.0 months; HR = 0.76, *p* < 0.001), PFS (5.2 vs. 1.9 months; HR = 0.44; *p* < 0.001), ORR (4% vs. 0.4%, *p* = 0.009) and DCR (64% vs. 48%, *p* < 0.001). These clinical outcomes were evaluated based on albumin–bilirubin (ALBI) grades in the CELESTIAL trial [40]. Further analysis of the Cabozantinib group indicated that the ALBI grade-1 subgroup had significantly better results than grade 2. Moreover, subgroup analysis showed that Cabozantinib was more efficient for those patients with extrahepatic spread, a high serum concentration of AFP (>400 ng/mL), or a good performance status (0–1) [72]. Patients with HBV tended to have a better result compared to HCV patients, which was more substantial with Lenvatinib compared to Sorafenib [53,60,72]. Patients aged ≥ 65, who were female or Western, benefited more from the treatment.

As previously described, almost 70% of patients had undergone only Sorafenib treatments before, while one-third of them had previously received two systemic treatments. For those patients who have received Sorafenib as the only previous therapy, Cabozantinib still prolonged the OS by almost 3 months [40,73]. Therefore, these data suggest that Cabozantinib is effective also as a second- or third-line treatment. As regards treatment strategy, the sequence of Sorafenib–Cabozantinib seems to improve PFS more than the Sorafenib–Regorafenib sequence, although with a similar OS between the two different sequences [74].

Grade ≥ 3 AEs occurred in 68% of patients in the experimental group and in 36% in the control group. The most frequent AEs deriving from Cabozantinib treatment were hypertension, palmar-plantar erythrodysesthesia (PPE), HFSR, diarrhea and fatigue [60].

As with Sorafenib and Regorafenib, a negative correlation existed between AEs and the survival data. In particular, patients that developed severe hypertension or PPE during Cabozantinib treatment showed better OS and PFS compared to those patients who did not experience these symptoms [73].

In addition, the CELESTIAL trial showed that a better prognosis during treatment with Cabozantinib was correlated with low levels of MET, GAS6, HGF, ANG2, VEGF-A, Interleukin (IL)-8, and high levels of insulin-like growth factor 1 in serum. However, these data have yet to be confirmed in clinical practice [75].

According to the CELESTIAL trial outcomes, Cabozantinib has been approved as a second-line therapy for advanced HCC patients.

#### 2.1.4. Regorafenib

Regorafenib (Stivarga) is a fluorinated analog of Sorafenib that is able to inhibit a greater number of tyrosine kinases: VEGFR-2, VEGFR-3, wild-type and mutant (V600E) B-RAF, KIT, PDGFR, RET, angiopoietin 1 receptor (TIE2), FGFR1, and p-38-alpha [76,77].

Regorafenib has greater anti-angiogenic and anti-proliferative activities compared to Sorafenib due to its greater potency to target VEGFR, TIE2, KIT, and RET [78]. It has been shown this TKI blocks cell growth and invasion, as well as reduces the expression of metastasis-related proteins in HCC cell lines [79]. Regorafenib can target the MAPK pathway, activate autophagy, and induce caspase cleavage [80,81]. Moreover, it also shows mitophagy activity. In this regard, the alteration of mitochondrial proteins, for example, BCL-xL, is related to Regorafenib resistance [82,83]. Furthermore, Regorafenib activates the intrinsic and extrinsic apoptotic pathways [84]. Regorafenib can overcome acquired resistance to Sorafenib, thanks to its ability to inhibit the activation of epithelial-mesenchymal transition (EMT) [85].

RESORCE is a double-blind, phase-III, randomized clinical trial that evaluated Regorafenib (*n* = 379) compared to placebo (*n* = 194) on 573 patients affected by advanced HCC, who progressed during Sorafenib treatment. All enrolled patients had radiological progression during Sorafenib treatment (at least 400 mg per day for 20 of the last 28 days) and good liver function (Child–Pugh Stage A). Approximately 40% of enrolled patients were from Western countries; hepatitis B was present in 38% of patients while 21% suffered from hepatitis C. Almost 70% presented extrahepatic disease and one-third had macrovascular invasion [41].

The results showed significant improvement of OS (10.6 vs. 7.8 months; HR = 0.63; *p* < 0.0001), PFS (3.1 vs. 1.5 months; HR = 0.46; *p* < 0.001) and TTP (3.2 vs. 1.5 months; HR = 0.44; *p* < 0.001). ORR (10.6% vs. 4.1%; *p* = 0.005) and DCR (65.2% vs. 36.1%; *p* = 0.001) were also improved, compared to placebo [41]. Further analyses documented the efficacy of Regorafenib in all subgroups, such as area of origin, serum AFP levels, and macrovascular invasion [41]. Moreover, patients who experienced an AFP response could obtain a survival benefit from this therapy compared to those patients without an AFP response [86,87]. Patients who were aged < 65, male, or Asian benefited more from the treatment.

Some plasma proteins, such as cystatin B, angiopoietin 1, oxidized low-density lipoprotein receptor 1, TGF-β, and C–C motif chemokine ligand 3 were found to be negatively associated with increased OS after Regorafenib treatment. Otherwise, plasma miRNAs (miR-30A, -122, -125B, -200A, -374B, -15B, -107, -320, and -645) were positively correlated with OS [88].

Severe AEs occurred in 44% of patients in the experimental group and in 47% of patients in the placebo group. Several AEs are similar to those for Sorafenib, including HFSR, hypertension, diarrhea, fatigue, increased bilirubin and serum aspartate aminotransferase (AST) levels, as well as their frequency, regardless of the last Sorafenib dose (800 mg/day) [41]. It is noteworthy that Regorafenib-related HFSR therapy is correlated with a prolonged OS, as seen during Sorafenib treatment [87]. A clinical study conducted by Kim et al. showed a poorer prognosis stage and a higher incidence of grade 3–4 AEs for patients with Child–Pugh Stage B during Regorafenib treatment compared to those with Child–Pugh Stage A [89].

Therefore, Regorafenib was approved as a second-line treatment for advanced HCC patients in progression during Sorafenib therapy.

### 2.2. VEGF Inhibitors

Experimental data demonstrated the pathogenetic role of angiogenesis in tumor growth [31,90,91,92,93,94,95,96,97,98,99,100]. As regards HCC, several studies showed the overactivation of VEGF and VEGFR signaling [101,102,103,104,105]. In support of these data, it has been described as the treatment with TKIs targeting the VEGF signaling pathway via multikinase inhibition that leads to a therapeutic benefit, albeit of modest size [11,27,31,33,35,36,93,103,106]. Hence, there is a necessity to further investigate alternative pathways targeting VEGF inhibition and tumor angiogenesis. Table 2 summarizes the VEGF inhibitors approved for the treatment of advanced HCC.

#### 2.2.1. Bevacizumab

Bevacizumab (Avastin) is an IgG humanized monoclonal antibody that prevents the activation of VEGFR by binding VEGF-A [103]. In this way, it blocks angiogenesis and tumor growth [103]. Several phase-II clinical studies have evaluated this drug in monotherapy and in combination with EGFR inhibitors or with chemotherapy, such as gemcitabine, capecitabine, and oxaliplatin [110,111,112,113,114,115,116]. The reported data showed that Bevacizumab was effective and safe. However, these studies suffered from some important limitations, including the small sample size and the absence of randomization. Therefore, no phase-III clinical trial was realized to confirm those results. Nowadays, several clinical researchers are evaluating the combination of anti-VEGF monoclonal antibodies and ICIs, with very interesting results. This new treatment strategy is based on the ability of anti-VEGF therapy to enhance the functions of effector T-cell and immune cell infiltration into TME and to blunt suppressive immune cells (Treg cells and MDSCs) [117,118,119,120]. Moreover, antiangiogenetic drugs augment tumor responsiveness to immunotherapy [120].

In this regard, the most impressive clinical trial is IMBrave150 [107]. This is a phase-III study that compared the combination of Atezolizumab (anti-PD-L1 antibody) and Bevacizumab (anti-VEGF antibody) with Sorafenib for advanced HCC patients. Approximately 60% of enrolled patients were from Western countries; hepatitis B was present in 49% of patients, while 21% suffered from hepatitis C. Almost 60% presented extrahepatic disease and 40% of them had a macrovascular invasion [107].

Nowadays, this combination of treatments is the only one able to significantly improve OS (19.2 vs. 13.4 months; HR = 0.66; *p* = 0.0009), PFS (6.8 months vs. 4.3 months; HR 0.59) and ORR (29.8% vs. 11.3%) as first-line treatment compared to Sorafenib [107]. In addition, combination therapy was demonstrated to enable these patients to receive long-term disease-free status (19 months) after surgical resection. Asian patients benefited more from this treatment.

As regards toxicity, hypertension and increased AST or ALT were the most common grade 3 or 4 AEs. However, no significant difference was observed regarding the risk of severe toxicity compared to the control group (56.5% vs. 55.1%). Moreover, a longer median time to the deterioration of QoL (11.2 vs. 3.6 months) was experienced by the experimental group [107]. However, Bevacizumab can cause bleeding, above all in cirrhotic patients, with a life-threatening risk; therefore, inclusion criteria included the evaluation of varices through upper gastrointestinal endoscopies and their treatment at least 6 months before the date of enrolment. Therefore, the administration of Atezolizumab plus Bevacizumab should be carefully evaluated in the case of patients at risk of bleeding, arterial hypertension, cardiovascular disease, and prior autoimmune conditions. Finally, patients with a low NLR value appear to experience a longer PFS compared to those with a high NLR (cumulative PFS at 150 days: 64% vs. 20%). Therefore, the NLR value before treatment might be considered a potential predictive factor of response to this treatment [107].

Interestingly, a recent study reported the utility of the C-reactive protein (CRP) and AFP in immunotherapy (CRAFITY) scores as predictive factors associated with PFS and OS in patients treated with Atezolizumab plus Bevacizumab. Moreover, this score could also predict treatment-related AEs [121].

Thanks to these very favorable results, IMbrave 150 was approved as a first-line therapy for advanced HCC patients without contraindications.

#### 2.2.2. Ramucirumab

Ramucirumab (Cyramza) is the only TKI that is administered intravenously. It is a recombinant monoclonal IgG1 antibody targeting VEGFR-2. In this way, Ramucirumab blocks ligand-receptor binding and the consequent downstream signaling [122].

REACH was a randomized, multicenter, phase III, clinical trial that analyzed Ramucirumab as a second-line therapy compared to a placebo for advanced HCC patients in progression during Sorafenib treatment [123]. Approximately 50% of enrolled patients were from Western countries; hepatitis B was present in 35% of patients, while 27% suffered from hepatitis C. Almost 70% presented extrahepatic disease and 30% of them had a macrovascular invasion [123].

The study did not show a statistically significant improvement of the primary endpoint (median OS 9.2 vs. 7.6 months; HR = 0.87; *p* = 0.14). The PFS was 2.8 months in the experimental group and 2.1 months in the control group (HR 0.63; *p* < 0.0001). ORR was 7% in the Ramucirumab group vs. <1% of the placebo group (*p* < 0.0001), while the DCR was 56% vs. 46% (*p* = 0.011), respectively [123]. Patients aged ≥ 65, who were female or from North and South America benefited more from the treatment.

The occurrence of Grade 3 or more AEs was higher in the Ramucirumab group (36%) compared to the control group (29%). The most common severe AE was malignant neoplasm progression [123].

Further subgroup analysis evidenced that this drug led to a greater benefit in OS for those patients with AFP ≥ 400 ng/mL than those with AFP < 0.0001, underling its potential role as a biomarker. Therefore, the phase III, double-blinded, randomized, REACH-2 clinical trial was designed to test the potential improvement of Ramucirumab for those patients who progressed to Sorafenib and who had a baseline AFP level of > 400 ng/mL compared to the placebo [109]. The study enrolled 292 advanced HCC patients, obtaining an OS of 8.5 months compared to 7.3 months of the placebo group (HR 0.710, 95% CI 0.531–0.949; *p* = 0.0199). As regards the secondary endpoints, PFS and DCR were 2.8 vs. 1.6 months (*p* < 0.0001) and 59.9% vs. 38.9% (*p* < 0.0006), respectively [109].

Severe AEs occurred in 35% and 29% of patients in the Ramucirumab group and in the placebo group, respectively. The most frequent severe AEs were hypertension, liver failure, and hyponatremia. Treatment discontinuation due to toxicity was observed in only 9.5% of patients in the experimental group [109].

A pooled analysis based on the REACH and REACH-2 trials also confirmed these results for advanced HCC patients with AFP ≥ 400 ng/mL, showing a higher benefit in the OS group vs. the placebo (OS 8.1 vs. 5.0 months; HR = 0.69; *p* = 0.0002) [124]. In addition, numerous subgroup analyses based on these clinical studies were designed to evaluate the relationship between the parameters of the Child–Pugh classification (such as ascites and ALBI grade) and outcomes for patients undergoing treatment with Ramucirumab. The data showed that the presence or absence of ascites had no impact on survival, while patients with ABLI Grade 1 or better had a longer OS than those with Grade 2 in the Ramucirumab arm [125,126,127].

According to these results, Ramucirumab was approved as a therapy for advanced HCC patients. Interestingly, it was the first drug for HCC that can be administered based on a biomarker level (AFP ≥ 400 ng/mL). However, the molecular mechanism explaining this selective action of Ramucirumab has not yet been well defined.

### 2.3. Other Targeted Agents

As is widely described, hepatocarcinogenesis and tumor progression consist of complex processes that depend on the crosstalk of several signaling pathways [128]. In light of this, numerous targeted molecules, both TKIs and non-TKIs, have been studied in the past few decades, although they have not yet been approved. Below is a brief description of the most significant ones.

#### 2.3.1. Tyrosine Kinase Inhibitors

Donafenib is a deuterated Sorafenib derivative that has been compared to Sorafenib as a front-line therapy for unresectable or metastatic HCC patients in a randomized multicenter, open-label, parallel-controlled phase II–III clinical trial [129]. The study enrolled 668 patients, from 37 centers across China, with a Child–Pugh score of ≤7 who had never been treated before. Approximately 50% of enrolled patients were from Western countries; hepatitis B was present in 90% of patients, while 2% suffered from hepatitis C. Almost 70% presented extrahepatic disease and/or macrovascular invasion [129].

The Donafenib group had a significantly improved mOS (12.1 months) compared to the control group (10.3 months; HR 0.831; *p* = 0.0245). Superior OS outcomes were also observed in the intention-to-treat population. The median PFS was similar (3.7 vs. 3.6 months; *p =* 0.0570), as well as the ORR (4.6% vs. 2.7%; *p* = 0.2448), and DCR (30.8% vs. 28.7%; *p* = 0.5532). Severe AEs occurred in a significantly lower percentage of the experimental group (38% vs. 50%; *p =* 0.0018) [129]. Therefore, Donafenib is a candidate to be a potential first-line therapy for these patients. However, an international multi-center clinical trial will be necessary to better evaluate this drug.

Nintedanib (BIBF 1120) is an oral triple angiokinase inhibitor of VEGFR, PDGFR, and FGFR. It was compared to Sorafenib in a randomized, multicenter, open-label, phase II study on 93 Asian patients with advanced HCC [130]. Hepatitis B was present in 63% of patients, while 14% suffered from hepatitis C. Almost 85% presented extrahepatic disease and/or macrovascular invasion. OS (11.9 vs. 11.4 months; HR 0.88) and TTP (5.5 vs. 3.8 months; HR 1.05) were comparable between the two groups. Severe AEs were more frequent in the Sorafenib group (68% vs. 90%), although treatment discontinuation due to AEs was higher with Nintetanib (45% vs. 23%). The most common AEs in the experimental group were diarrhea, vomiting, nausea, and AST increase [130].

Dovitinib, Vandetanib, Brivanib, and erlotinib showed low efficacy [131,132,133,134]. Linifanib (ABT-869) and sunitinib were responsible for unacceptable levels of toxicity [135].

See Table 3 for a summary of the other TKIs.

#### 2.3.2. Non-Tyrosine Kinase Inhibitors Targeting the Extracellular Space or Membrane

A series of non-RTKs is located in the cellular membrane, such as the receptors binding to tyrosine kinase transforming growth factor-beta (TGF-β), a serine/threonine kinase [136]. Dysregulation of the TGF-b signaling pathway has been seen in several cancers and is associated with tumor cell proliferation, migration, invasion, and cancer progression [137]. Therefore, the blockage of this pathway might present a viable strategy for the treatment of HCC.

In this regard, Galunisertib (LY2157299) is a small-molecule antagonist of TGF-β1 receptor type 1 (TGFBR1) with potential anticancer activity [138]. Galunisertib specifically binds to the kinase domain of TGFBR1, preventing the activation of the TGF-b-mediated signaling pathways. This may inhibit the proliferation of TGF-b-overexpressing tumor cells [139]. As documented in a phase II study, this drug led to a clinical benefit in advanced HCC patients with a lower baseline level of AFP as a second-line treatment [138]. Specifically, TTP was 2.7 months and 4.2 months for patients with a high baseline level of AFP (≥1.5× upper limit of normal) and lower AFP level, respectively. At the same time, the OS was 7.3 months and 16.8 months for patients with high baseline AFP levels and lower baseline AFP levels, respectively [137]. The OS was longer for those patients who experienced a decrease of > 20% from baseline (AFP responders), compared to non-responders (21.5 months vs. 6.8 months). Moreover, the OS was longer in TGF-β1 responders (>20% decrease from baseline) compared to non-responders. As regards toxicity, fatigue (33.6%), anemia (25.5%), peripheral edema (22.8%), and abdominal pain (21.5%) were the most common AEs. Neutropenia (2.7%) was the most frequent grade 3/4 AE [140].

Moreover, preclinical data suggest that Galunisertib might increase Sorafenib activity. Therefore, a phase II clinical trial evaluated the safety and efficacy of Galunisertib combined with Sorafenib as a front-line systemic treatment in 47 advanced HCC patients from 5 non-Asian countries with good liver function (Child–Pugh Stage A) [140]. The median TTP was 4.1 months, while the median OS was 18.8 months. The ORR was 4,5%, and the DCR was 51%. Patients who experienced a TGF-β1 decrease of > 20% from baseline (TGF-β1 responders) demonstrated a longer OS (22.8 vs. 12.0 months; *p* = 0.038) compared to non-responders. Severe AEs occurred in 59.6% of patients [140].

Moreover, numerous glycoproteins placed in the cellular membrane could be promising treatment targets. For example, Endoglin (CD105) is a co-receptor for TGF-β and is involved in tumor angiogenesis, inflammation, and fibrogenesis [141]. An anti-CD105 monoclonal antibody (TRC105) was evaluated in some studies, demonstrating its ability to inhibit tumor angiogenesis [142]. Moreover, it led to a significant ORR (25%) in combination with Sorafenib for the treatment of HCC. The median PFS was 3.8 months, while the median OS was 15.5 months.

In fact, a multi-center Phase-II clinical trial is ongoing to better evaluate this effect.

See Table 4 for a summary of the inhibitors targeting these areas.

#### 2.3.3. Agents Targeting the Intracellular Space

Numerous intracellular signaling pathways may play pivotal roles in tumor development. For example, it has been widely reported in the literature that the PI3K/AKT/mTOR and Ras/Raf/MAPK pathways are involved in vascular invasion, intrahepatic metastasis, and Sorafenib resistance [143,144]. Therefore, new drugs targeting the intracellular pathways have been studied. SF1126 acts to inhibit the PI3K/BRD4 and Ras/Raf/MAPK pathways [145]. It has been tested as a single agent and in combination with Sorafenib, demonstrating significant antitumor activity in vivo [146].

As regards mTOR inhibitors, Everolimus was analyzed in a randomized multi-center, multinational phase-II trial as a single agent or in combination with Sorafenib and showed a modest survival benefit, despite its ability to reverse Sorafenib resistance [147]. EVOLVE-1 was a randomized, double-blind, phase-III study conducted on advanced HCC patients who progressed during or after Sorafenib, or who were intolerant of Sorafenib (*n* = 362), compared to the placebo (*n* = 184) [148]. Approximately 60% of the enrolled patients were from Asian countries; hepatitis B was present in 26% of patients, while 25% suffered from hepatitis C. Almost 74% presented extrahepatic disease and 33% had a macrovascular invasion. No significant difference in OS was observed between the two groups (7.6 vs. 7.3 months; HR 1.05; *p* = 0.68). The median TTP was 3.0 and 2.6 months, respectively (HR, 0.93). DCR was 56.1% and 45.1%, respectively (*p* = 0.01). Severe AEs were more frequent in the experimental group (70.9% vs. 52.2%, respectively). The most frequent grade 3/4 AEs for Everolimus were anemia, asthenia, and decreased appetite [148].

Selumetinib and Refametinib are MEK inhibitors that have been demonstrated to have a synergistic effect with Sorafenib in the treatment of advanced HCC. Selumetinib led to an ORR of 15%, while the most common AEs were diarrhea, rash, and hypertension [149]. Refametinib, as a single agent, documented an ORR of 0% and a DCR of 56.3%. The OS was 5.8 months, while the PFS was 1.9 months [150]. The combination of Refametinib with Sorafenib led to an ORR of 6.3% and a DCR of 43.8%. The OS was 12.7 months, and the PFS was 1.5 months. Fatigue, hypertension, and acneiform rash were the most common AEs [150].

Moreover, it has been demonstrated that Sorafenib has a synergistic effect, with numerous inhibitors targeting nuclear signaling molecules, as described in preclinical studies, including Ribociclib (cyclin-dependent kinase 4/6 inhibitors), and Palbociclib (PD-0332991). In addition, Resminostat (a histone deacetylase inhibitor) was tested as a single agent and in combination with Sorafenib as a second-line therapy in the SHELTER study [151]. This trial documented a PFS rate after 6 treatment cycles of 12.5% for the single agent and a rate of 62.5% for the combination treatment. The TTP and OS were 1.8 and 4.1 months for Resminostat and 6.5 and 8.0 months for Resminostat plus Sorafenib, respectively. The most common AEs were gastrointestinal disorders, thrombocytopenia, and fatigue [151].

Finally, CT-707 (YAP signaling inhibitor) and OPB-111077 (STAT3 inhibitor) were tested on in vivo or in vitro HCC models but showed only limited efficacy.

See Table 5 for a summary of the described durgs.

## 3. Conclusions

Important progress has been made in the therapeutic strategy of advanced HCC during the last 15 years, starting with Sorafenib, which was approved in 2007, and continuing up to the other new treatment options (Lenvatinib and Atezolizumab-Bevacizumab) as first-line therapies for these patients. Cabozantinib, Regorafenib, and Ramucirumab (AFP > 400 ng/mL) are the current therapeutic options for second-line treatment.

The experimental data demonstrated that hepatocarcinogenesis and tumor progression consist of complex processes, depending on the crosstalk of several signaling pathways. In light of this finding, numerous targeted molecules, TKIs, and non-TKIs have been studied in the last few decades, although their use has not yet been approved. Interestingly, some of them have shown encouraging results. In this regard, the identification of biomarkers can help to identify special HCC populations who might better benefit from the different targeted therapies.

Finally, based on the remarkable results reported in terms of OS, derived from the association of atezolizumab plus bevacizumab, novel combinations of ICIs and TKIs are already being applied in clinical trials. 

## Figures and Tables

**Table 1:** Tyrosine kinase inhibitors for the treatment of advanced HCC.

TKI	Trial	Comparison	Setting	Clinical Features of Enrolled Patients (%)	OS	PFS/TTRP *	ORR/DCR **	Grade 3–4 AEs
Sorafenib (Nexavar)	SHARP (Phase III) [37]	Placebo	First-line	- Child–Pugh Stage A (97%), Westerns - HCV (29%), alcohol intake, HBV (19%) - Extrahepatic disease (51%) - Macroscopic vascular invasion (70%)	10.7 vs. 7.9 months; HR = 0.69; *p* < 0.001	5.5 vs. 2.8 months; HR = 0.58; *p* < 0.001 *	43% vs. 32%; *p* = 0.002 **	8% vs. 2%; *p* < 0.001
Sorafenib (Nexavar)	ASIAN PACIFIC (Phase III) [38]	Placebo	First-line	- Child–Pugh Stage A (97%), Orientals - HBV (71%), HCV (11%) - Extrahepatic disease (69%) - Macroscopic vascular invasion (35%)	6.5 vs. 4.2 months; HR = 0.68; *p* = 0.014	2.8 vs. 1.4 months; HR = 0.57; *p* = 0.0005	35% vs. 16%; *p* = 0.0019 **	9% vs. 1%
Lenvatinib (Lenvima)	REFLECT (Phase III) [39]	Sorafenib	First-line	- Child–Pugh Stage A (99%) - Westerners (30%), Asians (70%) - HBV (53%), HCV (19%) - Extrahepatic disease (60%) - No ≥ 50% liver tumor burden, gross invasion of the bile duct or the main portal vein	13.6 vs. 12.3 months; HR = 0.92	7.4 vs. 3.7 months; HR 0.66; *p* < 0.0001	24.1%vs. 9.2%; *p* < 0.0001 75% vs. 60%	75% vs. 67%
Cabozantinib (Cometriq, Cabometyx)	CELESTIAL (Phase III) [40]	Placebo	Second-/Third-line	- Child–Pugh Stage A (98%), Westerns (70%) - HBV (38%), HCV (25%) - Extrahepatic disease (80%) - Macrovascular invasion (30%)	10.2 vs. 8.0 months; HR = 0.76; *p* <0.001	5.2 vs. 1.9 months; HR = 0.44; *p* < 0.001	4% vs. 0.4%; *p* = 0.009/ 64% vs. 48%; *p* < 0.001 **	68% vs. 36%
Regorafenib (Stivarga)	RESORCE (Phase III) [41]	Placebo	Second-line	- Child–Pugh Stage A (98%), Orientals (40%) - HBV (38%), alcohol intake (25%), HCV (21%) - Extrahepatic disease (70%) - Macrovascular invasion (30%)	10.6 vs. 7.8 months; HR = 0.63; *p* < 0.0001	3.1 vs. 1.5 months; HR = 0.46; *p* < 0.001; 3.2 vs. 1.5 months; HR = 0.44; *p* < 0.001 *	10.6% vs. 4.1%; *p* = 0.005 65.2% vs. 36%; *p* = 0.001	44% vs. 47%

**Abbreviations:** tyrosine kinase inhibitor (TKI); Progression free-Survival (PFS); Overall Survival (OS); Objective Response Rate (ORR); Time to Radiological Progression (TTRP) *; Disease Control Rate (DCR) **.

**Table 2:** VEGF Inhibitors for treatment of advanced HCC.

VEGF Inhibitor	Trial	Comparison	Setting	Enrolled Patients (%)	OS	PFS	ORR/DCR *	Grade 3–4 AEs
Bevacizumab (Avastin) + Atezolizumab (Tecentriq)	IMBrave150 (Phase III) [107]	Sorafenib	First-line	- Child–Pugh Stage A (99%) - Westerns (60%) - HBV (49%), HCV (21%). - Extrahepatic disease (60%) - Macrovascular invasion (40%) - Untreated or incompletely treated esophageal or gastric varices (excluded)	19.2 vs. 13.4 months; HR = 0.66; *p* = 0.0009	6.8 vs. 4.3 months; HR 0.59	29.8% vs. 11.3%	56.5% vs. 55.1%
Ramucirumab (Cyramza)	REACH (Phase III) [108]	Placebo	Second-line	- Child–Pugh Stage A (98%) - Westerns (50%) - HBV (35%), HCV (27%). - Extrahepatic disease (70%) - Macrovascular invasion (30%)	9.2 vs. 7.6 months; HR = 0.87, *p* = 0.14	2.8 vs. 2.1 months; HR 0.63; *p* < 0.0001	7% vs. <1%; *p* < 0.0001 56% vs. 46%; *p* = 0.011 *	36% vs. 29%
Ramucirumab (Cyramnza)	REACH-2 (Phase III) [109]	Sorafenib	Second-line	- Child–Pugh Stage A (100%) - Orientals (50%) - HBV (36%), HCV (24%) - Extrahepatic disease (70%) - Macrovascular invasion (35%) - Baseline AFP level of > 400 ng/mL	8.5 vs. 7.3 months HR 0.710, *p* = 0.0199	2.8 vs. 1.6 months *p* < 0.0001	59.9% vs.38.9% *p* < 0.0006	35% vs. 29%

**Abbreviations:** Vascular Endothelial Growth Factor (VEGF); Progression free-Survival (PFS); Overall Survival (OS); Objective Response Rate (ORR); Disease Control Rate (DCR) *.

**Table 3:** Other tyrosine kinase inhibitors for the treatment of advanced HCC.

TKI	Trial	Comparison	Setting	Enrolled Patients (%)	OS	PFS	ORR/DCR *	Grade 3–4 AEs
Donafenib (Zepsun)	Phase II–III [129]	Sorafenib	First-line	- Child–Pugh stage A (97%) - HBV (90%), HCV (2%). - Extrahepatic disease and/or Macrovascular invasion (70%)	12.1 vs. 10.3 months HR 0.831; *p* = 0.0245	3.7 vs. 3.6 months; *p =* 0.0570	4.6% vs. 2.7%, *p* = 0.02448 30.8% vs. 28.7%; *p* = 0.5532 *	38% vs. 50%; *p =* 0.0018
Nindetanib (BIBF 1120)	Phase II [130]	Sorafenib	First-line	- Child–Pugh Stage A (99%) - Orientals (100%) - HBV (63%), HCV (14%). - Extrahepatic disease and/or Macrovascular invasion (85%)	11.9 vs. 11.4 months; HR 0.88	5.5 vs. 3.8 months; HR 1.05		68% vs. 90%

**Abbreviations:** Tyrosine kinase inhibitor (TKI); Progression free-Survival (PFS); Overall Survival (OS); Objective Response Rate (ORR); Disease Control Rate (DCR) *.

**Table 4:** Non-tyrosine kinase inhibitors targeting the extracellular space or membrane.

Non-TKI	Trial	Comparison	Setting	Enrolled Patients (%)	OS	PFS/TTRP *	ORR/DCR **	Grade 3–4 AEs
Galunisertib (LY2157299)	Phase II [139]	/	Second-line	- Child–Pugh Stage A (100%) - Westerns (85%) - HBV (20%), HCV (24%) - Macrovascular invasion (26%)	7.3 months for patients with high baseline AFP levels 16.8 months for patients with lower baseline AFP levels	2.7 months for patients with high baseline AFP levels * 4.2 months for patients with lower baseline AFP levels *	2%	43.6%
Galunisertib (LY2157299) + Sorafenib (Nexavar)	Phase II [140]	/	First-line	- Child–Pugh Stage A (100%) - Westerns (46%) - HBV (18%), HCV (34%). - Extrahepatic disease (80%) - Macrovascular invasion (34%)	18.8 months	4.1 months *	4.5% 51% **	59.5%
TRC105 + Sorafenib (Nexavar)	Phase I [142]	/	First-line	- Child–Pugh Stage A (90%) - HBV (12%), HCV (60%). - Extrahepatic disease (68%)	15.5 months	3.8 months	25%	52%

**Abbreviations:** tyrosine kinase inhibitor (TKI); Progression free-Survival (PFS); Overall Survival (OS); Objective Response Rate (ORR); Time to Radiological Progression (TTRP) *; Disease Control Rate (DCR) **.

**Table 5:** Agents targeting the intracellular space.

Drug	Trial	Comparison	Setting	Enrolled Patients (%)	OS	PFS/TTRP *	ORR/DCR **	Grade 3–4 AEs
Everolimus (Afinitor)	VOLVE-1 (Phase III) [148]	Placebo	Second-line	- Child–Pugh Stage A (100%) - Orientals (60%) - HBV (26%), HCV (25%) - Extrahepatic disease (74%) - Macrovascular invasion (33%)	7.6 vs. 7.3 months (HR 1.05; *p* = 0.68)	3.0 vs. 2.6 months (HR 0.93) *	56.1% vs. 45.1% (*p* = 0.01)	70.9% vs. 52.2%
Selumetinib	Phase I/II [149]	/	First-line	- Child–Pugh Stage A (96.3%) - Orientals (60%) - HBV (59.3%), HCV (7.4%) - Extrahepatic disease (48%) - Macrovascular invasion (26%)	/	/	15%	Diarrhea, rash, hypertension
Refametinib Refametinib + Sorafenib	Phase II [150]	/	First-line	- Child–Pugh Stage A (100%) - Orientals (56%) - HBV (12%), HCV (6%) - Extrahepatic disease (50%) - Macrovascular invasion (35%)	5.8 months 12.7 months	1.9 months 1.5 months	0% 56.3% ** 6.3% 43.8% **	Fatigue, hypertension, and acneiform rash
Resminostat Resminostat + Sorafenib	SHELTER (Phase I/II)	/	Second-line	- Child–Pugh Stage A (100%) - Westerns (100%) - HBV (32%), HCV (15%). - Extrahepatic disease (60%)	4.1 months 8 months	1.8 months * 6.5 months *	/	Nausea (11%), asthenia (11%) Thrombocytopenia(12%), diarrhea (8%), hypertension (8%)

**Abbreviations:** Progression free-Survival (PFS); Overall Survival (OS); Objective Response Rate (ORR); Time to Radiological Progression (TTRP) *; Disease Control Rate (DCR) **.
